# Calculation of a conversion factor for estimating the glycolytic contribution in exercise from post-exercise blood lactate concentration

**DOI:** 10.3389/fphys.2023.1283327

**Published:** 2024-01-24

**Authors:** David W. Hill, John Michael Mihalek

**Affiliations:** ^1^ Applied Physiology Laboratory, University of North Texas, Denton, TX, United States; ^2^ College of Applied Human Sciences, West Virginia University, Morgantown, WV, United States

**Keywords:** anaerobic, cycling, energy demand, exercise intensity, glycolysis, heavy intensity

## Abstract

**Purpose:** Often, the glycolytic contribution in a bout of heavy or severe intensity exercise is estimated by multiplying the increase in blood lactate concentration above resting levels that is engendered by the exercise (in mM) by 3.3 (or 3) mL·kg^−1^ per mM. Our purpose was to verify the value of this conversion factor, using methods that were completely different from those of the original studies.

**Methods:** Six women (mean ± SD), age, 23 ± 1 year; VO_2max_, 46 ± 4 mL·kg^−1^·min^−1^) and three men (23 ± 0 years; 54 ± 8 mL·kg^−1^·min^−1^) completed 6 min of heavy intensity exercise in conditions of normoxia and hypoxia (F_I_O_2_, ∼12%). VO_2_ was measured throughout the exercise and 7 min of recovery. The increase in glycolytic contribution was estimated as the reduction in aerobic contribution in hypoxia, after correction for the effects of hypoxia on the oxygen demand and on the contribution from phosphocreatine. The peak post-exercise blood lactate concentration was measured in fingerstick blood samples.

**Results:** The ratio between the increase in estimated glycolytic contribution (in mL·kg^–1^) in hypoxia and the increase in peak blood lactate concentration (in mM) yielded an oxygen equivalent of 3.4 ± 0.4 mL·kg^–1^ per mM (range, 2.6 mL·kg^−1^ per mM to 4.0 mL·kg^−^1 per mM) for cycle ergometer exercise.

**Conclusion:** These results generally support the use of a common conversion factor to calculate the glycolytic contribution from post-exercise blood lactate concentrations. However, there is some inter-individual variability in the conversion factor.

## Introduction

It is often important to quantify the anaerobic contribution during exercise or to quantify the *maximal* possible contribution, which is the anaerobic capacity. Maximal accumulated oxygen deficit (MAOD) is the gold standard measure of anaerobic capacity (Medbø et al., 1988; Noordhof et al., 2010). Unfortunately, its calculation requires contentious assumptions, and it is time-consuming (Bangsbo, 1996; Hill et al., 2002; Özyener et al., 2003). An alternative method, popularized by Bertuzzi and colleagues (2010), calculates the alactic or phosphocreatine (PCr) contribution from the post-exercise oxygen uptake (VO_2_) profile (Margaria et al., 1933; Henry, 1951) and calculates the glycolytic contribution using the peak post-exercise blood lactate concentration (PeakLAC) (Margaria et al., 1933), specifically using the ‘net’ or ‘accumulated’ blood lactate, which is calculated as PeakLAC minus the resting concentration. The sum of the lactic and alactic contributions, which has been called ‘*PCr + glycolysis’*, ‘*MAOD-alternative’,* or *‘MAOD_alt’*, reflects the total anaerobic contribution. This method is attractive because it requires only one test (Bertuzzi et al., 2010), it generates separate and independent measures of the alactic and lactic contributions (Bertuzzi et al., 2010), and it can be used to generate an intensity-specific and mode-specific measure of efficiency (Valenzuela et al., 2020). *MAOD_alt* has been used in literally dozens of studies (*e.g.,* De Campos Mello et al., 2009; Zagatto and Gobatto, 2012; Milioni et al., 2016; Zagatto et al., 2016; de Poli et al., 2019). However, there is very little experimental evidence to support the methods used to estimate the lactic contribution.

We will note here that, like the highest attainable aerobic rate or maximal VO_2_ (VO_2max_), the greatest possible anaerobic contribution or anaerobic capacity (MAOD) is mode-specific. For example, higher values for both VO_2max_ and MAOD are obtained in running compared to cycling (*e.g.,* Hill and Vingren, 2011). In this paper, we used cycle ergometer exercise and we shall refer to our values as ‘maximal’.

The oxygen equivalent of blood lactate (O_2_-equivalent–lactate) is the conversion factor that is used to calculate glycolytic contribution from blood lactate concentration. Its value was first estimated by Margaria and colleagues (1933), based on the presumed stoichiometry of glycolysis and estimations of the rate of lactate production, distribution, and/or accumulation. They reported a value of 3.3 mL·kg^−1^ per mM of blood lactate.

Ferretti (2015) has argued that, while “the energy equivalent of blood lactate concentration … remains a key concept of extreme utility in the analysis of whole-body energy expenditure, … our current knowledge still relies mostly on the classical results obtained by the School of Margaria between the sixties and eighties of the last century” (pp. 158, 159). So true. In fact, there has been only one study (Gladden and Welch, 1978) that has attempted to validate or test or determine the value of the O_2_-equivalent–lactate using methods that did not rely on the assumptions and methods used by the School of Margaria.

If the O_2_-equivalent–lactate is going to be used with confidence to calculate *MAOD-alt* or to calculate the glycolytic contribution alone, then clearly it must be experimentally confirmed to be valid (as attempted in the study by Gladden and Welch, 1978). The purpose of the present study was to calculate the value of the O_2_-equivalent–lactate conversion factor or, in other words, to validate the value that was calculated by Margaria and colleagues in their classic 1933 paper. The design of our study is similar to that of the study published by Gladden and Welch (1978), and the approach is completely different from that of the early studies performed in the School of Margaria. In this study, we calculated directly the value of O_2_-equivalent–lactate using measures of glycolytic contribution and the associated PeakLAC.

## Methods

### Participants

The procedures were approved by the Institutional Review Board for Protection of Human Subjects in Research at the university, and the study was conducted in accordance with the *Declaration of Helsinki* (World Health Organization, 2014). The participants were nine healthy university students, all Kinesiology majors, among them six women (mean [SD], age, 23 ± 1 year; height, 166 ± 7 cm; weight, 67 ± 8 kg) and three men (age, 23 ± 0 years; height, 178 ± 3 cm; weight, 80 ± 7 kg). They provided written consent after the procedures, risks, and benefits of the study had been explained. All participants were involved in recreational sport or fitness activities, but none was a highly trained athlete. They verified that they did not change their exercise routines, diet, or sleep habits over the course of the study.

After they had provided consent, potential participants were screened using the Physical Activity Readiness Questionnaire (PAR-Q; Thomas et al., 1992) and a brief medical history. Individuals were excluded based on responses to the PAR-Q or if they were a smoker, had donated blood or plasma in the preceding 2 months, or reported an illness. Prior to data collection, as part of pilot testing, all participants performed several incremental tests and severe intensity tests to exhaustion, during which the fraction of oxygen in the inspired air (F_I_O_2_) was manipulated.

### Design

A descriptive research design was used to identify the mathematical relationship between quantitative measures of glycolytic contribution and blood lactate accumulation. For each participant, preliminary testing included an incremental exercise test. Then, each participant performed two constant power tests, one in normoxia (F_I_O_2_, ∼21%) and one in hypoxia (F_I_O_2_, ∼12%). Each test was terminated after 6 min so that the oxygen cost in the two tests would be comparable, although estimably higher in hypoxia. For each participant, both the glycolytic contribution and the PeakLAC were increased in hypoxia. We calculated the ratio between the increase in glycolytic contribution and the increase in PeakLAC. This ratio represents the O_2_-equivalent–lactate (in mL·kg−1 per mM) (see Eq. 1).

Testing was performed in a climate-controlled laboratory (21.6°C ± 0.5°C, 40% ± 4% relative humidity) and there were no differences in temperature and humidity across the two conditions. Sessions were separated by 2–8 days. Each participant’s tests were scheduled at the same time of day, in order to avoid the confounding effects of circadian rhythmicity (Hill, 2014).

### Incremental tests to determine VO_2max_

These tests were performed in normoxia on an electronically braked Lode Excalibur (Groningen, Netherlands) cycle ergometer. Pedalling cadence was 80 rev·min^–1^. Initial work rate was 50 W (30 W for one participant who weighed 53.8 kg). Stages were 3 min in duration and increments were 20 W–30 W, individually selected by the senior investigator. After the fourth stage, durations were reduced to 1 min and increments were increased to 40 W–70 W. The highest work rate that was sustained for 1 min was recorded as the peak power.

Expired gases were analyzed using a MedGraphics (St. Paul, MN, United States) UltimaO_2_ system, which was calibrated before each test. Breath-by-breath VO_2_ data were reduced to serial 15-s averages, and VO_2max_ was the highest average of adjacent 15-s values. Heart rate (HR) and peripheral capillary oxygen saturation (S_p_O_2_) were monitored using an Innovo Medical iP900AP monitor (Stafford, TX, United States), and a rating of perceived exertion (RPE; Borg, 1970) was obtained near the end of each stage. Tests were terminated when pedalling cadence dropped below 70 rev·min^–1^ for 5 seconds, despite strong verbal encouragement.

### Constant power tests

There was 4 min of seated rest, 4 min of moderate intensity warm-up, 4 min of seated rest, 6 min of heavy intensity exercise, and 7 min of seated recovery. Only data from the heavy intensity exercise and the recovery were used. The work rates were individually selected, based on responses during the incremental tests, to be near the upper end of the heavy intensity domain. Typically, it was ∼50% of the peak power. The work rates for the warm-up and heavy intensity exercise were 48 ± 7 W (0.7 ± 0.1 W·kg^−1^) and 137 ± 22 W (1.9 ± 0.4 W·kg^−1^), respectively.

### Control of F_I_O_2_ during constant power tests

Air from three MAG-20 generators (Higher Peak, Stoneham, MA, United States), which were connected in parallel, was fed *via* a 50-L expansion/buffer bag to the mouthpiece. In the first 30 s of exercise, flow rate and oxygen extraction were adjusted until F_I_O_2_ was close to the desired value (21% or 12%).

### Measurement of PeakLAC

After the heavy intensity exercise, the participant sat quietly. Blood samples were obtained from a warmed fingertip 4 min, 5 min, and 6 min into recovery and analyzed in duplicate using identical Accusport Lactate analysers (Hawthorne, NY, United States). The highest value was PeakLAC.

### Calculation of O_2_-equivalent–lactate

The *conceptual* framework for calculations is presented here. The *practical* description of methods begins in the following paragraph. *Conceptually*, the calculation involves three assumptions: i) the energy cost (oxygen cost) of exercise is the same in hypoxia and normoxia, ii) the aerobic contribution can be quantified by the area under the curve of the VO_2_ response (AUC_total), and iii) the decrease in aerobic contribution in hypoxia is compensated by an increase in glycolytic contribution of equal magnitude. So, the ratio between the decrease in aerobic contribution in hypoxia (the increase in glycolytic contribution, in mL·kg^−1^) and the increase in PeakLAC (in mM) equals the O_2_-equivalent–lactate (in mL·kg^−1^ per mM).

As noted above, these three assumptions do not accurately describe the exercise scenario. First, because part of the oxygen cost of exercise is the cost of ventilation, and a greater ventilatory response is engendered in hypoxia, the increase in anaerobic contribution must include the extra cost of ventilation (ΔVO_2,VENT_); its calculation is described below.

Second, oxygen demand (in mL·kg^−1^·min^−1^) actually increases during a bout of heavy exercise, in concert with the expression of the slow component of the VO_2_ response; so, differences in the slow component contribution in hypoxia versus normoxia might also have to be considered in calculating the increase in anaerobic contribution in hypoxia. However, if the anaerobic contribution (oxygen deficit) is the difference between the total oxygen cost (in mL·kg^−1^) (which includes the excess oxygen uptake associated with the slow component) minus the total area under the VO_2_ response curve (in mL·kg^−1^) (which also includes the excess oxygen uptake associated with the slow component), then the excess oxygen uptake associated with the slow component “cancels out”. Calculation of the pertinent measure of aerobic contribution, the area under the curve of the primary phase of the VO_2_ response (AUC_primary), is described below.

The third assumption was that the reduction in aerobic contribution is compensated by an increase in glycolytic contribution. However, part of the compensatory response might possibly include an increase in PCr contribution (not *just* glycolysis). Therefore, the increase in anaerobic contribution in hypoxia must exclude any increase in PCr contribution (ΔPCr); its calculation is described below (see Calculation of ΔPCr for use in Eq. 1).

With these assumptions and considerations, the calculation of the O_2_-equivalent–lactate is (Eq. 1):

$$O_{2}-\text{equivalent}–\text{lactate}=\left(\left(\text{AUC}\_{\text{primary}}_{\text{normoxia}}\;-\text{ AUC}\_{\text{primary}}_{\text{hypoxia}}\right)+\Delta {\text{VO}}_{2,\text{VENT}}\;-\;\Delta \text{PCr}\right)\;/\left({\text{PeakLAC}}_{\text{hypoxia}}\;-\;{\text{PeakLAC}}_{\text{normoxia}}\right)$$ (1)

**FIGURE 1:**
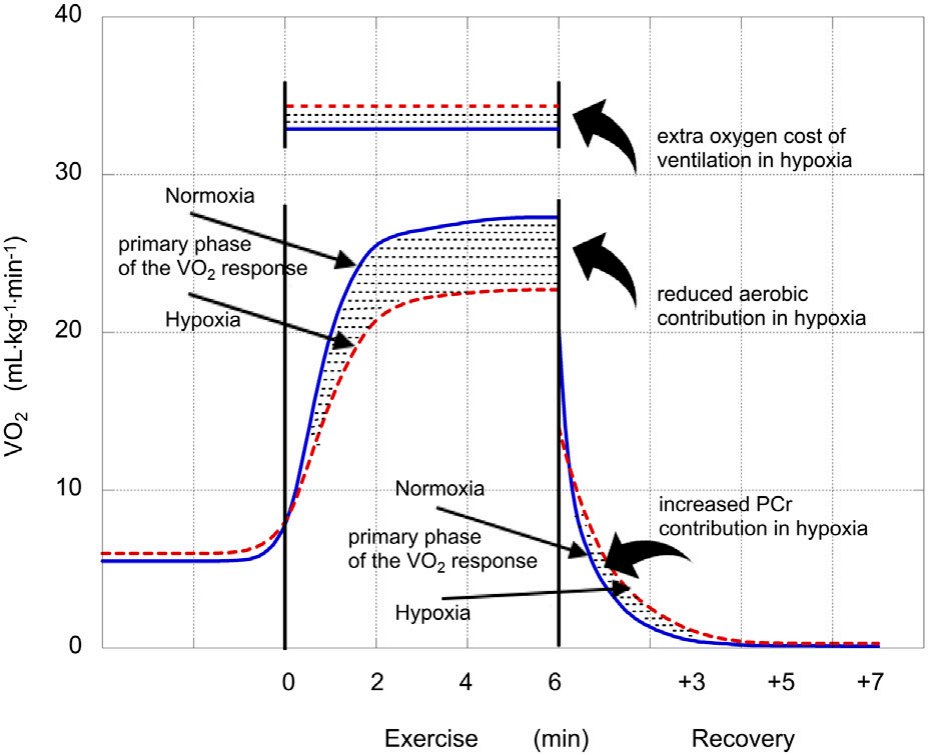
Illustration of the VO_2_ responses in normoxia (solid blue lines) and hypoxia (dashed red lines). Straight arrows identify the phases of the VO_2_ responses (mL·kg^−1^·min^−1^) and stylistic arrows identify oxygen costs or contributions (mL·kg^−1^) that were affected by hypoxia. The reduced aerobic contribution during exercise performed in hypoxia was compensated by an increased anaerobic contribution. The increase in glycolytic contribution in hypoxia is calculated as the sum of that increase plus the extra oxygen cost of ventilation in hypoxia minus the increase in PCr contribution in hypoxia (Eq. 1).

This scenario is presented in Figure 1, using mean values from this study. These VO_2_ curves reflect the baseline-plus-primary-response, not the overall VO_2_ response. So, in hypoxia, the curve levels off at 27.3 mL·kg^−1^·min^–1^, not at the end-exercise value of 30.4 mL·kg^−1^·min^–1^. The stippled area between the two curves describes the greater anaerobic contribution in hypoxia; the stippled rectangular area across the top represents the excess oxygen demand associated with the exaggerated ventilatory response (ΔVO_2,VENT_); and the stippled area between the two curves in recovery represents ΔPCr.

### Calculation of AUC_primary for use in Eq. 2

Breath-by-breath VO_2_ data were reduced to 5-breath averages. Data from the first 20 s were removed (Murias et al., 2011) and parameters of the VO_2_ response profile were determined using nonlinear regression in KaleidaGraph 4.50 (Reading, PA, United States), fitting data to Eq. 2:

$${\text{VO}}_{2}\left(t\right)=A_{\text{baseline}}+A_{\text{primary}}\times \left(1\;–\;e^{–\;\left(\left(\;t\;–\;\text{TDprimary}\;\right)\;/\;\text{tauprimary}\;\right)}\right)+A_{\text{slow}}\times \left(1\;–\;e^{–\;\left(\left(\;t\;–\;\text{TDslow}\;\right)\;/\;\text{tauslow}\;\right)}\right)$$ (2)

VO_2_(t) is the value for VO_2_ at time = t; A_baseline_ is VO_2_ prior to exercise; A_primary_ and A_slow_ are the asymptotic amplitudes for the primary and slow terms, respectively; tau_primary_ and tau_slow_ are the time constants; and TD_primary_ and TD_slow_ are the time delays before expression of the responses. Values for AUC_total and AUC_primary were calculated using integration in KaleidaGraph.

### Calculation of ΔPCr for use in Eq. 1

The post-exercise breath-by-breath VO_2_ data were reduced to rolling 5-breath averages. The parameters of the VO_2_ response profile were determined using KaleidaGraph and Eq. 3:

$${\text{VO}}_{2}\left(t\right)=A_{\text{baseline}}+A_{\text{fast}}\times (e^{–\;(\;t\;/\;\text{taufast})}+A_{\text{slow}}\times \left(e^{–\;\left(\;t\;–\;\text{TD}\right)\;/\;\text{tauslow}}\right))$$ (3)

VO_2_(t) is the value for VO_2_ at time = t; A_baseline_ is the VO_2_ prior to exercise; A_fast_ and A_slow_ are the asymptotic amplitudes; and tau_fast_ and tau_slow_ are the time constants. The area under the curve of the fast phase of the response represents the PCr contribution in the exercise (Margaria et al., 1933; Henry, 1951; Gladden and Welch, 1978)). The ΔPCr was calculated as the PCr contribution in hypoxia minus the PCr contribution in normoxia.

### Calculation of the ΔVO_2,VENT_ in hypoxia for use in Eq. 1

Vella et al., 2006 calculated the oxygen cost of breathing at ∼2.1 mL per L of ventilation, at ∼50% V_Emax_, and that it increases exponentially with greater V_E_. We estimated the extra cost of ventilation in hypoxia (ΔVO_2,VENT_) as 2.5 mL per L of extra ventilation.

### Statistical analyses

Data were collapsed across sex for statistical analyses. Results of the Shapiro-Wilk test revealed that the data were normally distributed. The responses in normoxia and hypoxia were compared using a paired-means *t*-test in SPSS v.24 (IBM, Armonk, NY, United States). The correlation between the increase in glycolytic contribution in hypoxia and the increase in PeakLAC (numerator and denominator in Eq. 1 was calculated. Individual values for the ratio between the increase in glycolytic contribution in hypoxia and the increase in PeakLAC (numerator and denominator in Eq. 1 The mean value was compared to 3.2 ± 0.4 mL·kg^−1^ per mM (Gladden and Welch, 1978) using an independent-samples *t*-test and it was compared with a range of fixed values that bracketed the mean using single-sample *t*-tests. Significance for all analyses was set at *p* < 0.05. Data are reported as mean ± SD.

## Results

### Incremental tests to determine VO_2max_

The mean 30-s VO_2max_ was 46 ± 4 mL·kg^−1^·min^−1^ for women, 54 ± 8 mL·kg^−1^·min^−1^ for men, and 49 ± 5 mL·kg^−1^·min^–1^ overall. Peak power was 269 ± 67 W (3.7 ± 0.5 W·kg^−1^). Other max values were: HR, 176 ± 8 bt·min^−1^; RER, 1.12 ± 0.03; and RPE, 20 ± 1.

### Selected responses during the constant power tests

Mean work rate was ∼51% of peak power; end-exercise VO_2_ was ∼62% of VO_2max_ in normoxia and ∼58% of VO_2max_ in hypoxia. End-exercise HR was 21 ± 5 bt·min^−1^ higher (*p* < 0.01) in hypoxia (165 ± 8 bt·min^−1^) than normoxia (144 ± 12 bt·min^−1^); end-exercise RPE was 1 ± 1 higher (*p* < 0.01) in hypoxia (14 ± 1) than in normoxia (13 ± 1).

### O_2_-equivalent–lactate

Pertinent responses during exercise in hypoxia, in Table 1, included the reduction in (causing greater reliance on anaerobic pathways in hypoxia), the increase in V_E_ (causing an increased oxygen demand in hypoxia), and the increase in the area under the curve of the fast phase of the recovery VO_2_ (reflecting a slightly greater PCr contribution in hypoxia).

**TABLE 1:** Factors impacted by hypoxia and used in the calculation of the oxygen equivalent of peakLAC (O_2_-equivalent–lactate). Difference scores are absolute values. Values are mean [SD].

Variable	Normoxia	Hypoxia	Difference	t-test
VO_2baseline_ (mL·kg^−1^) (95% CI)	5.4 ± 0.8 (4.9, 5.9)	6.0 ± 0.7 (5.5, 6.5)	0.6 ± 0.2 (0.5, 0.7)	p < 0.01
A_primary_ (mL·kg^−1^) (95% CI) SEE^a^	21.9 ± 4.4 (19.0, 24.8) 0.3 ± 0.1	16.7 ± 3.2 (14.6, 18.8) 0.7 ± 0.8	5.2 ± 0.4 (5.5, 5.0)	p < 0.01
TD_primary_ (s) (95% CI) SEE^a^	5 ± 2 (4, 6) 2 ± 1	7 ± 4 (4, 10) 3 ± 2	2 ± 6 (–2, 6)	p = 0.36
tau_primary_ (s) (95% CI) SEE^a^	30 ± 5 (26, 33) 3 ± 1	28 ± 8 (23, 34) 2 ± 2	2 ± 7 (–2, 7)	p = 0.46
MRT_primary_ (s) (95% CI)	35 ± 6 (31, 39)	35 ± 9 (29, 41)	0 ± 4 (–3, 3)	p = 0.89
AUC_primary_ (mL·kg^−1^) (95% CI)	149.4 ± 22.9 (134.4, 164.4)	126.5 ± 19.7 (111.4, 137.2)	22.9 ± 7.4 (16.7, 29.1)	p < 0.01
PeakLAC (mM) (95% CI)	4.9 ± 1.0± (4.2, 5.5)	12.6 ± 2.3 (11.1, 14.1)	7.7 ± 2.4 (6.1, 9.3)	p < 0.01
V_E_ ^b^ (L·min^−1^) (95% CI)	55 ± 8 (50, 60)	80 ± 13 (72, 89)	25 ± 5 (21, 30)	p < 0.01
ΔVO_2VENT_ (mL·kg^−1^) (95% CI)			5.0 ± 1.0 (4.3, 5.7)	p < 0.01^c^
Recovery A_fast_ (mL·kg^−1^) (95% CI) SEE^a^	20.3 ± 3.2 (18.2, 22.4) 2 ± 1	15.2 ± 2.8 (13.4, 17.0) 2 ± 2	5.1 ± 1.5 (4.1, 6.1)	p < 0.01
Recovery tau_fast_ (s) (95% CI) SEE^a^	38 ± 4 (35, 41) 3 ± 1	57 ± 10 (50, 63) 3 ± 5	19 ± 4 (16, 22)	p < 0.01
PCr (mL·kg^−1^) (95% CI)	12.8 ± 1.6 (11.8, 13.9)	14.4 ± 2.4 (12.8, 16.0)	1.6 ± 1.8 (0.4, 2.8)	p = 0.04
VO_2_ ^b^ (mL·kg^–1^·min^−1^) (95% CI)	30.4 ± 4.8 (27.3, 33.5)	28.1 [4.5 (25.2, 31.0)	2.3 [1.0 (1.7, 3.0)	p < 0.01
HR^b^ (bt·min^−1^) (95% CI)	144 ± 12 (136, 152)	165 ± 8 (160, 170)	21 ± 5 (18, 24)	p < 0.01
O_2_-equivalent–lactate (95% CI)	3.4 ± 0.4 mL·kg^−1^ per mM (3.1 mL·kg^−1^ per mM, 3.7 mL·kg^−1^ per mM)			

^a^ A standard error of the estimate (SEE) is associated with each parameter estimate that was generated by KaleidaGraph; smaller SEE, reflect greater confidence in the accuracy of the estimate.

^b^ 30-s value from the end of the 6 min of exercise.

^c^ Single sample *t*-test comparing mean difference against zero.

The increases in glycolytic contribution and the increases in PeakLAC in hypoxia were strongly correlated (*r* = 0.95, *p* < 0.01). Mean O_2_-equivalent–lactate was 3.4 ± 0.4 mL·kg^−1^ per mM (range, 2.6 mL·kg^−1^ per mM to 4.0 mL·kg^−1^ per mM). This was not different (*p* = 0.33) from the 3.2 ± 0.4 mL·kg^−1^ per mM, reported for four participants by Gladden and Welch (1978), or the 3.3 mL·kg^−1^ per mM (no SD) estimated by Margaria and colleagues (1933) (*p* = 0.53). It was different (at *p* < 0.05) from any value that was ≤3.0 mL·kg^−1^ per mM or ≥3.8 mL·kg^−1^ per mM.

While the study was not powered to evaluate the effect of sex, we note that the women’s value was 3.4 ± 0.4 m L·kg^−1^ per mM and the men’s was 3.4 ± 0.2 mL·kg^−1^ per mM.

## Discussion

The important finding was that the glycolytic contribution in cycle ergometer exercise can be estimated using the PeakLAC after exercise, using 3.4 ± 0.4 mL·kg^−1^ per mM for the conversion. The homogeneity of O_2_-equivalent–lactate values (95% CI, 3.1 mL·kg^−1^ per mM, 3.7 mL·kg^−1^ per mM) suggests that a common conversion factor may be appropriate. However, the inter-individual variability in values indicates that some caution must be exercised. The propriety of using a common value for O_2_-equivalent–lactate depends on its intended use.

One rationale for this study is that researchers are using values of either 3.0 mL·kg^−1^ per mM or 3.3 mL·kg^−1^ per mM in the calculation *PCr + glycolysis* as a measure of anaerobic capacity and *assuming* that value of this conversion factor is accurate (*e.g.,* De Campos Mello et al., 2009; Zagatto and Gobatto, 2012; Milioni et al., 2016; Zagatto et al., 2016; de Poli et al., 2019). The *PCr + glycolysis* (or *MAOD-alt*) method is attractive, because it requires only one severe intensity exercise test, performed to exhaustion. In contrast, maximal accumulated oxygen deficit, the gold standard measure of anaerobic capacity (Medbø et al., 1988; Noordhof et al., 2010), requires several moderate intensity bouts in addition to the severe intensity test, and it requires several questionable assumptions (Bangsbo, 1996; Hill et al., 2002; Özyener et al., 2003; Noordhof et al., 2010), including that the VO_2_—work rate relationship is linear and that the oxygen demand of constant power exercise remains constant. In fact, we have shown that the VO_2_—work rate relationship may be upwardly curvilinear (Hill and Vingren, 2011) and that the oxygen demand increases over the course of supra-threshold exercise (Riojas et al., 2020). Calculations in the present study did not rely on any estimation of the oxygen demand because it did not require measurement of the total anaerobic contribution (oxygen deficit). The second rationale for this study is based on the arguments from Ferretti (2015). While the methods used in the present study are simplistic, and cannot provide any depth of information about the important topic of anaerobic bioenergetics in exercise, they do directly address the question of the validity of using *PCr + glycolysis* as an indicator of anaerobic capacity.

Early estimations of the O_2_-equivalent–lactate (Margaria et al., 1933; 1963) measured PeakLAC and assumed that “*a*) [lactate] is freely and rapidly diffusing uniformly in all the water of the body … and *b*) that the fraction of water was 0.6 for the body as a whole, and 0.8 for the blood” (Margaria et al., 1963, p.372) to estimate the amount of lactate that had been produced during exercise. Then, using a “caloric equivalent” of lactate of 222 cal·g^−1^, the O_2_-equivalent–lactate could be expressed as 3.3 mL·kg^−1^ per mM. Clearly, the production, distribution, and removal of lactate are very complex processes, as has been well documented (Brooks, 1985; 2009). The underlying assumption in estimating glycolytic contribution is that processes that remove lactate before it enters the blood–such as its utilization as a fuel in the muscles–are consistent enough among individuals that he resulting concentration of lactate in the blood provides quantitative evidence of the lactate production. A detailed description of the significance of blood lactate measures and the distribution of lactate in the body during severe intensity exercise is also provided by Ferretti (2015), chapters three and six.

Gladden and Welch (1978) had two women and six men perform a series of cycle ergometer exercise tests in varying degrees of hypoxia. For these participants, the O_2_-equivalent–lactate was 5.2 mL·kg^−1^ per mM. However, they noted that, “based on standard errors of the estimates [for individual participants’ results], it is obvious that this ratio could easily vary from 3.0 to 8.4” (Gladden and Welch, 1978, p. 567). For the four men with a significant linear relationship between PeakLAC and F_I_O_2_, the O_2_-equivalent–lactate was 3.2 ± 0.4 mL·kg^−1^ per mM. Suggesting a possible weakness of their study, the authors commented that, although V_E_ was higher when F_I_O_2_ was lower, they did not consider the possible impact on oxygen demand. Had they considered this, the estimates of the O_2_-equivalent–lactate would have been higher. They also did not consider possible differences in the PCr contributions; however, they did estimate the PCr contributions by back-extrapolating the VO_2_ from minute 6 to minute 15 of recovery, calculating the area under this regression line, and subtracting this area from the post-exercise VO_2_ curve, and they reported that PCr contributions were independent of F_I_O_2_. In contrast, we report a slightly higher PCr contribution in hypoxia, and we calculated ΔPCr directly, by modeling the recovery response.

A range of values for O_2_-equivalent–lactate have been reported–from 2.7 mL·kg^−1^ per mM to 3.3 mL·kg^−1^ per mM (Ferretti, 2015). For example, di Prampero et al. (1978) reported a value 0f 2.7 mL·kg^−1^ per mM in swimming and Margaria’s group (1963) reported ∼3.0 mL·kg^−1^ per mM for three men in running. These results suggest that the value for O_2_-equivalent–lactate may be mode-specific. Of note, the calculations assumed that water fractions of the blood and whole body are 0.8 and 0.6, respectively, that lactate is uniformly distributed, that 222 cal/g is the energy release from lactate. It is altogether possible that the numbers are not exactly 0.8 and 0.6 (*i.e.,* that hematocrit demonstrates inter-individual variability), and that lactate is not absolutely uniformly distributed. In addition, despite that the value for energy release from lactate is assumed to be 222 cal/g, authors have calculated it to be either 235 cal/g (Margaria et al., 1971) or 250 cal/g (Margaria et al., 1963). Had these latter values been used in calculations, then the estimates of O_2_-equivalent–lactate would have been 6%–13% higher … actually greater than the value that we calculated. So, while one might certainly question the 3.4 mL·kg^−1^ per mM that was experimentally determined in the present study, it might also be fair to ask if the assumptions used in early studies were 100% accurate for all individuals and all populations. In this paper, we neither rely on nor question the assumptions. However, the similarity of values obtained in this study using completely different methods supports that those assumptions may be valid representations of population means.

The study of the energetics of heavy, severe, and extreme intensity exercise is vitally important and yet “the study of the energetics of anaerobic supramaximal exercise underwent a decay in recent years, so that our current knowledge still relies mostly on the classical results obtained by the School of Margaria between the sixties and eighties of last century” (Ferretti, 2015, p. 158). In the present study, we addressed his concern about reliance on these classic studies, but our study was not without weaknesses. With only nine participants, we could not hope to identify the true value of the O_2_-equivalent–lactate (if there is one) and we cannot suggest if there are *different* values for O_2_-equivalent–lactate that would be more suitable for use with different populations (*e.g.,* young versus old, trained versus untrained). Future studies could include a larger and more diverse subject population. It is also true that we focussed on the relationship between PeakLAC and glycolytic contribution and made no attempt to contribute to the body of knowledge regarding bioenergetics; it is of utmost importance that future research addresses the needs compellingly identified by Ferretti (2015).

Using appropriate values for O_2_-equivalent–lactate, sport scientists and coaches can easily and rapidly measure the glycolytic capacity in the laboratory as well as in the field, for example, after competitions (in which the truest value of glycolytic capacity can be elicited). In addition, during training sessions, the method allows immediate measurement of glycolytic contribution for assessment of the demands of the exercise and can be used ‘on the spot’ to assess the suitability of the training methods and to modify the training program.

## Conclusion

In this study, the value of the O_2_-equivalent–lactate for heavy intensity cycle ergometer exercise was calculated to be 3.4 ± 0.4 mL·kg^−1^ per mM. These results support the use of a common conversion factor to calculate the glycolytic contribution, which can be used by itself or to calculate *PCr + glycolysis*. The results also demonstrate inter-individual variability in the value for O_2_-equivalent–lactate, which indicates that some caution must be exercised in its application.

## Data Availability

The raw data supporting the conclusion of this article will be made available by the authors, without undue reservation.
